# Data set of the proteome of fresh and frozen thawed stallion spermatozoa

**DOI:** 10.1016/j.dib.2020.105887

**Published:** 2020-06-20

**Authors:** Francisco E. Martín-Cano, Gemma Gaitskell-Phillips, José M. Ortiz-Rodríguez, Antonio Silva, Cruz Gil, Cristina Ortega-Ferrusola, Fernando J. Peña

**Affiliations:** a Laboratory of Equine Reproduction and Spermatology, Department of Animal Medicine, Veterinary Teaching Hospital, University of Extremadura, Cáceres, Spain; b SIPA University of Extremadura, Cáceres, Spain

**Keywords:** Spermatozoa, Proteomics, Horses, Cryopreservation

## Abstract

This paper provides the dataset of proteins of stallion ejaculates before and after cryopreservation. The data report the analysis and identification of stallion sperm proteins obtained from the same ejaculates and split in two subsamples. The first aliquot consisted on fresh spermatozoa and the second aliquot was frozen and thawed spermatozoa. Samples were analyzed using a UHPLC/MS/MS system consisting of an Agilent 1290 infinity series UHPLC coupled to an Agilent 6550 Q-TOF mass spectrometer (Agilent Technologies, Santa Clara, CA, USA). We provide a data set of 2226 different proteins, with 2180 aligned to the equine proteome database. The data can be used to identify potential targets to be explored to improve techniques for cryopreservation of spermatozoa. This data article refers to the article “Proteomic profiling of stallion spermatozoa suggests changes in sperm metabolism and compromised redox regulation after cryopreservation” (Martín Cano et al.; 2020) [1].

**Specifications Table**

**Table:** 

**Subject**	Veterinary Medicine and Veterinary Science; Reproductive Biology.
**Specific subject area**	Proteomics of stallion spermatozoa.
**Type of data**	Raw data
**How data were acquired**	Samples were analyzed using a UHPLC/MS/MS system consisting of an Agilent 1290 infinity series UHPLC coupled to an Agilent 6550 Q-TOF mass spectrometer (Agilent Technologies, Santa Clara, CA, USA)
**Data format**	Processed Analyzed
**Parameters for data collection**	Fresh and frozen thawed equine spermatozoa using a split sample design from the same ejaculates.
**Description of data collection**	Comparative mass spectrometry proteomic profiling of fresh and frozen thawed equine spermatozoa
**Data source location**	Cáceres, Extremadura, Spain
**Data accessibility**	Data are available via the PRIDE partner repository with the dataset identifier PRIDE PXD018111. **ProteomeXchange title:** changes induced by cryopreservation in horse spermatozoa **ProteomeXchange accession:** PXD018111 **PubMed ID:**32247875 **Project Webpage:** http://www.ebi.ac.uk/pride/archive/projects/PXD018111 **FTP Download:** ftp://ftp.pride.ebi.ac.uk/pride/data/archive/2020/04/PXD018111
**Related research article**	Francisco E Martín Cano, Gemma Gaitskell-Phillips, Jose M. Ortíz Rodríguez, Antonio Silva, Cruz Gil, Cristina Ortega Ferrusola, Fernando J Peña (2020). Proteomic profiling of stallion spermatozoa suggests changes in sperm metabolism and compromised redox regulation after cryopreservation *J. Proteom.* 221:103,765 doi.org/10.1016/j.jprot.2020.103765

**Value of the Data**

Proteomic data reported here can be useful for cryobiologists and spermatologists. The data can be used to identify signaling pathways affected by the procedure and potential targets to be monitored to improve current cryopreservation protocols.
In addition, this data can be used to identify important signaling pathways involved in stallion sperm biology
The whole dataset of proteins can be used in diverse applications including as a guide to identify biomarkers of stallion sperm quality and potential fertility
The data reported point to new lines of research for improvement of cryopreservation technologies. The target pathways and proteins provided here can help to identify specific steps in the procedure (cooling, toxicity of cryoprotectants etc..) which can be modified to reduce damage.

## 1 Data description

A data set of 2203 proteins obtained from equine spermatozoa and its changes after cryopreservation is provided. This information could lead to a better understanding of cryodamage in the spermatozoa. The data set has been deposited to the ProteomeXchange Consortium [2] via the PRIDE partner repository with the dataset identifier PRIDE PXD018111.

## 2 Experimental design, materials, and methods

### 2.1 Sample preparation

Spermatozoa were washed three times in PBS (600 gx 10′) and samples of stallion semen before and after cryopreservation were pelleted and stored at −80 °C until processing and analysis. To assure the absence of debris and other contaminating cells in the samples visual inspection using phase contrast microscopy was performed. Before preparation for proteomic analysis individual fresh and thawed samples from each stallion were pooled to generate 6 different pools, with the aliquots of semen before and after cryopreservation obtained from the same stallion and ejaculate. Then samples containing 200 × 10^6^ cells were processed as described in [1]. The first step was solubilization of proteins in a buffer consisting of C7:C7Bz0 [3-(4-heptyl) phenyl-(3-hydroxypropyl) dimethylammoniopropanesulfonate, 7 M urea, 2 M thiourea and 40 mM Tris (pH 10.4). To achieve this goal, 20 microliters of lysis buffer was added per every 10 × 10^6^ spermatozoa. The solution formed was thoroughly vortexed and then incubated under constant rotation at a temperature of −4 °C for 60 min. To measure the quantity of protein obtained in the previous step, the 2-D Quant Kit (GE Healthcare, Sevilla Spain) was used. All the samples were normalized to a final concentration of 100 μg of protein. In the next step, 200 µL of the solution of protein obtained were mixed with 100 μl of 25 mM ammonium bicarbonate buffer pH 8.5 (100 μg of protein in 300 μL of solution). Addition of 30 µL of 10 mM DTT and incubation for 20 min at 56° was used to reduce the proteins in the sample. In the next step alkylation of proteins was performed by the addition of 30 µL of 20 mM IAA solution, and incubation for 30 min at room temperature in the dark. Finally, proteins were digested adding 1 μL of Trypsin Proteomics Grade (Sigma) (Trypsin solution: 1 μg/μL in 1 mM HCl) for a period of at least 3 h to overnight at a temperature of 37 °C. To stop this reaction 10 μL of 0.1% formic acid was added. Then the solution was filtered through 0.2 μm (hydrophilic PTFE) into 2 ml vials made of dark glass. In the last step samples were dried using a nitrogen flow with the vial in a heating block at 35 °C. Then 20 μl of buffer A, consisting of water/acetonitrile/formic acid (94.9:5:0.1) was added to the dry samples to resuspend the proteins

### 2.2 UHPLC-MS/MS analysis

We processed the samples as described in [1] using a UHPLC/MS system, comprising an Agilent 1290 Infinity II Series UHPLC (Agilent Technologies, Santa Clara, CA, USA) provided with an automated multisampler module and a High Speed Binary Pump, coupled to an Agilent 6550 Q-TOF Mass Spectrometer (Agilent Technologies, Santa Clara, CA, USA) with an Agilent Jet Stream Dual electrospray (AJS-Dual ESI) interface. Both systems HPLC and Q-TOF were controlled by the Mass Hunter Workstation Data Acquisition software (Agilent Technologies, Rev. B.06.01). The injection of the samples was done at a flow rate of 0.4 ml/min onto an Agilent AdvanceBio Peptide Mapping HPLC column (2.7 μm, 150 × 2.1 mm, Agilent technologies), at 55 °C. The gradient analysis was initiated with 2% of Buffer B (water/acetonitrile/formic acid, 10:89.9:0.1) in isocratic mode for 5 min. The flow posteriorly increased linearly up to 45% of Buffer B over 40 min, and then increased again to 95% over a period of 15 min. Then the flow remained constant for further 5 min. After this first run of 70 min of duration, we conditioned the column for the next run for 5 min using the initial condition. We operated the mass spectrometer in positive mode, and we set to 35 psi the nebulizer gas pressure. The temperature of the gas was 250 °C and the flow rate was set to 10 l/min with the sheath gas set at 300 °C at a flow rate of 12 L/min. The voltages of the capillary spray, fragmentor and octupole VR were set a 3500, 340 and 750 V respectively. The extended dynamic range mode was used to acquire profile data for MS and MS/MS scans. The mass ranges for MS and MS/MS were 50–1700 *m/z* while the scan rates were 8 and 3 spectra per second for MS and MS/MS respectively. Precursor selection by abundance in auto MS/MS mode was used, with 20 precursors per cycle as a maximum. A slope of 3–6 and an offset of −4.8 was used in the ramped collision energy. Finally, after two consecutive scans the same ion was discharged.

### 2.3 Data processing

The Spectrum Mill MS Proteomics Workbench (Rev B.04.01, Agilent Technologies, Santa Clara, CA, USA) was used for data processing and analysis as described in [1]. The raw data were extracted as follows; we selected non fixed modifications; [MH]+ 50–10,000 *m/z*; maximum precursor charge +5; retention time and *m/z* tolerance ± 60 s; minimum signal-to-noise MS (S/N) 25; finding ^12^C signals. The MS/MS search was done against the updated Uniprot/Horse protein database and was conducted as using the following criteria: non fixed modifications and the following variable modifications were selected: carbamidomethylated cysteines, tryptic digestion with the maximum missed cleavages set at 5. The ESI-Q-TOF instrument was set to the following criteria; minimum matched peak intensity 50%, maximum ambiguous precursor charge +5, monoisotopic masses, mass tolerance for peptide precursor 20 ppm, mass tolerance for product ion 50 ppm and calculation of reversed database scores. We validated peptide and protein data using auto thresholds with a% false discovery rate (FDR) of 1.2%. The result for proteins was obtained as protein summarized using all validations, score >4 and% Scored Peak Intensity (SPI) that is defined as the percentage of the extracted spectrum that is explained by the database search result, set at 60.

## Declaration of Competing Interest

The authors declare that they have no known competing financial interests or personal relationships which have, or could be perceived to have, influenced the work reported in this article.

## Appendix Supplementary materials

Image, application 1
